# Delayed Ureteral Obstruction Due to Intramural Endo-Endoscopic Gastrointestinal Anastomosis Staples Following Laparoscopic Sigmoid Resection: A Case Report

**DOI:** 10.7759/cureus.98395

**Published:** 2025-12-03

**Authors:** Zsolt Madarasz, Bogdan-Cornel Sturzu, Krzysztof Nowakowski, Ewald Becker, Jens Hoeppner, Julia Michel, Anas Baltamar

**Affiliations:** 1 Department of Surgery, Medical School and University Medical Center OWL, Campus Lippe, Bielefeld University, Detmold, DEU; 2 Department of Urology, Medical School and University Medical Center OWL, Campus Lippe, Bielefeld University, Detmold, DEU

**Keywords:** delayed ureteral obstruction, flexible ureterorenoscopy, foreign body urolithiasis, intramural staple, laparoscopic colorectal surgery, surgical stapler complication, ureteral injury

## Abstract

Ureteral injuries are among the most significant urological complications associated with colorectal surgery. Although most iatrogenic ureteral injuries are detected intraoperatively or shortly after surgery, a considerable number are identified only later. Very delayed manifestations that occur months or even years after the initial procedure are uncommon and pose diagnostic challenges. We report a case of a 50-year-old female patient who presented with intermittent abdominal discomfort and was found to have a left-sided mid-ureteral stone with grade I hydronephrosis. Her surgical history included an elective laparoscopic sigmoid resection six years earlier for a covered perforation secondary to sigmoid diverticulitis, during which an Endo-Gastrointestinal Anastomosis (GIA) stapler was used. Ureterorenoscopy with laser lithotripsy revealed two metallic staples, one embedded in the ureteral wall, and another encased within the stone matrix, both successfully removed endoscopically. Postoperative retrograde ureteropyelography and renal ultrasound confirmed resolution of the obstruction without residual stricture. To our knowledge, this represents the first documented case of delayed ureteral obstruction caused by retained intramural and intraluminal Endo-GIA staples. The presumed mechanism involves inadvertent staple incorporation during pelvic dissection, followed by chronic foreign body reaction and subsequent stone formation. Endoscopic treatment proved effective, avoiding the need for reconstructive surgery. This case highlights the importance of meticulous ureteral identification during pelvic procedures and supports the selective use of visualization-enhancing techniques in high-risk surgical settings.

## Introduction

Iatrogenic ureteral injuries are uncommon but potentially serious complications of pelvic and colorectal surgery, and their incidence is strongly influenced by the surgeon’s experience and the complexity of the operative field. Reported incidence rates range from 0.3% to 1.5% in elective abdominal operations, underscoring that even minimally invasive approaches carry inherent risk [1,2]. A large retrospective analysis of the National Surgical Quality Improvement Program (NSQIP) database found an incidence of approximately 0.6%, reinforcing the clinical relevance of this complication [3].

Laparoscopic sigmoid resection performed in the setting of complicated diverticulitis poses a particularly high risk for ureteral injury due to inflammation, adhesions, and distorted anatomical planes. Prior studies have shown that laparoscopic colectomy is associated with a higher incidence of ureteral injury compared to open procedures, especially when diverticulitis is the indication [3]. Other investigations have highlighted the increased risk of iatrogenic ureteral injury in laparoscopic colorectal surgery performed under severely inflamed conditions, as well as the potential benefits of intraoperative fluorescent ureteral catheter techniques in reducing this risk [4,5].

Although most ureteral injuries are recognized intraoperatively or in the early postoperative period, delayed presentations, occurring months or even years after the index procedure, are exceedingly rare and diagnostically challenging. Surgical staplers such as the Endo-Gastrointestinal Anastomosis (GIA) system are routinely used in minimally invasive procedures due to their efficiency and reliability. However, inadvertent inclusion of adjacent anatomical structures, including the ureter, may occur, particularly in cases involving distorted pelvic anatomy [3,6]. While direct evidence of stapler-induced ureteral injury remains limited, similar complications have been reported in the context of inflammation, reoperative fields, or altered anatomy [2,5,6].

Foreign body-induced urolithiasis is another well-documented phenomenon in urology. Stones have been reported to form around migrated surgical clips, non-absorbable sutures, or retained ureteral stents left in situ for extended periods, often leading to obstruction or infection [7-9]. However, the formation of urinary calculi around retained surgical staples represents an exceptionally rare and previously undocumented cause of ureteral obstruction.

To our knowledge, this appears to be the first documented case of delayed ureteral obstruction caused by retained Endo-GIA staples, one embedded within the ureteral wall and the other incorporated into a ureteral calculus, diagnosed and successfully managed six years after laparoscopic sigmoid resection.

## Case presentation

A 50-year-old female patient with a history of obesity (BMI 40 kg/m²) presented in June 2025 with persistent right lower quadrant abdominal discomfort. Her medical history included an elective laparoscopic sigmoid resection performed in June 2019 for sigmoid diverticulitis, classified as Hinchey Ib [10]. The initial episode had been treated conservatively with antibiotics, and definitive surgery was scheduled in an inflammation-free interval after a colonoscopy had excluded malignancy. The procedure was carried out by a junior attending surgeon who had recently completed specialist training but already possessed considerable experience in laparoscopic colorectal surgery. Intraoperatively, moderate inflammatory adhesions between the sigmoid colon and the left pelvic sidewall were noted, but the operation proceeded without documented complications. An Endo-GIA stapling device was used for both transection and anastomosis. The postoperative course was uneventful, and the patient was discharged on postoperative day four. Additional surgical history included laparoscopic cholecystectomy, multiple cesarean sections, and childhood appendectomy.

Clinical examination revealed localized tenderness in the right lower quadrant without guarding, rebound, or signs of peritonitis. Initial laboratory work-up revealed a mildly elevated C-reactive protein (CRP) of 10.7 mg/dl (reference value: <0.5 mg/dl), while the white blood cell count was within normal limits. Renal function and urinalysis were unremarkable.

**Figure 1:**
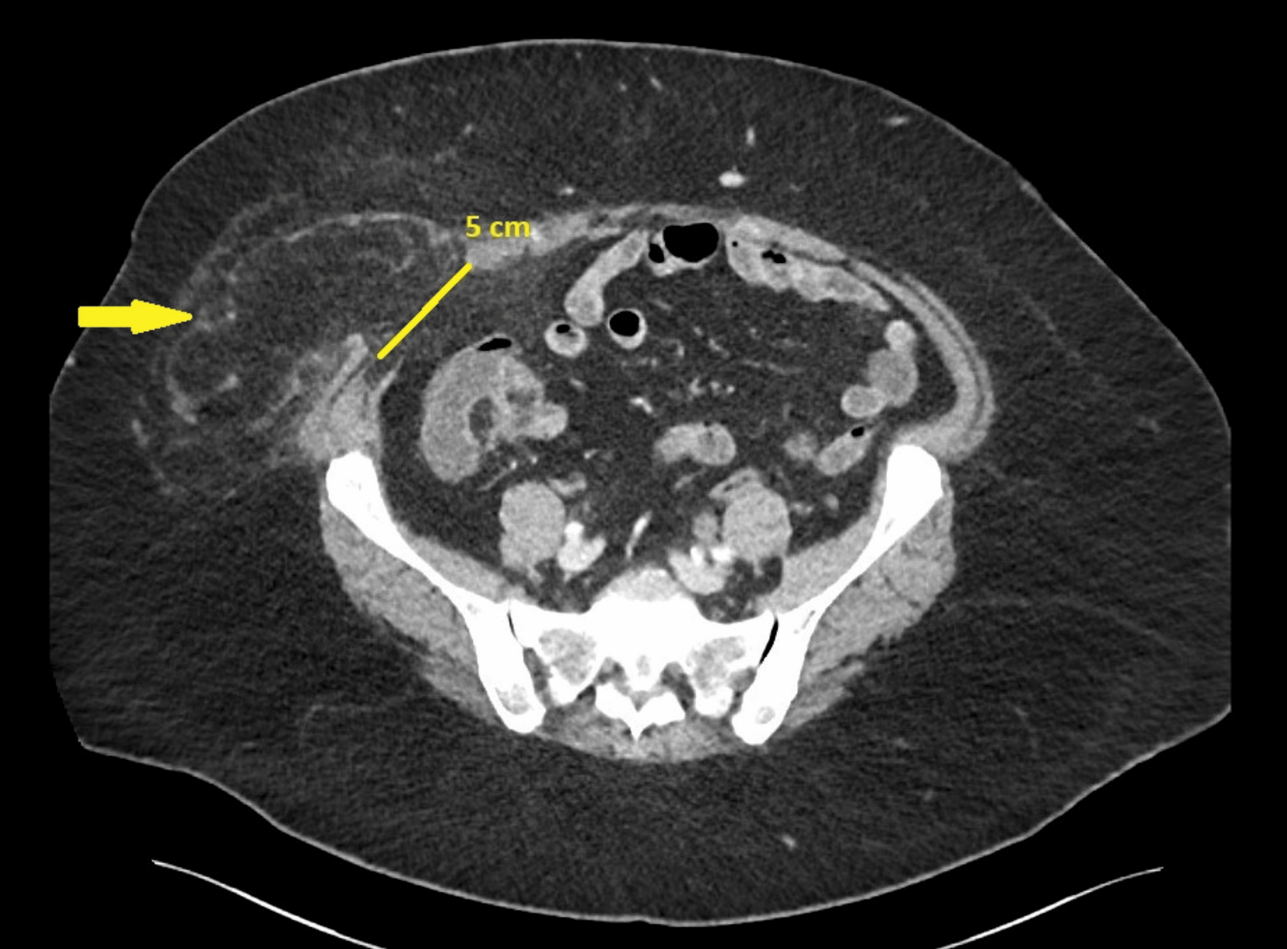
Axial CT scan demonstrating a right lower quadrant incisional hernia Axial CT image of the abdomen and pelvis showing a right lower quadrant incisional hernia (~5 cm) at the previous bowel extraction site from the 2019 laparoscopic sigmoid resection. The hernia sac contains omental fat without evidence of bowel incarceration. The yellow arrow indicates the location of the incisional hernia.

Contrast-enhanced computed tomography (CT) imaging of the abdomen and pelvis revealed two significant findings: 1) A right-sided incisional hernia (Figure 1) with a 50 mm fascial defect, containing omental fat, without evidence of bowel incarceration; and

**Figure 2:**
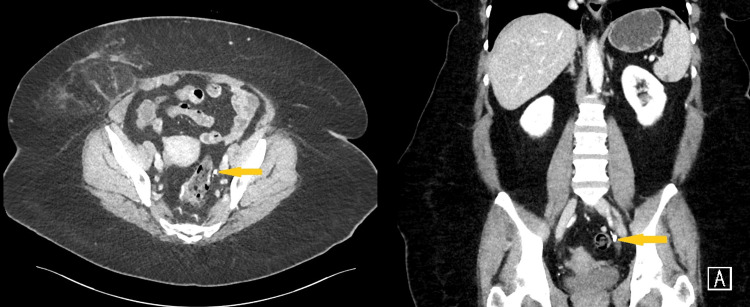
Combined axial and coronal CT imaging demonstrating a left-sided mid-ureteral calculus Axial (left) and coronal (right) contrast-enhanced CT images of the abdomen and pelvis. The yellow arrows indicate a hyperdense calculus located in the mid-portion of the left ureter at the level of the pelvic brim, a common site of ureteral narrowing.

**Figure 3:**
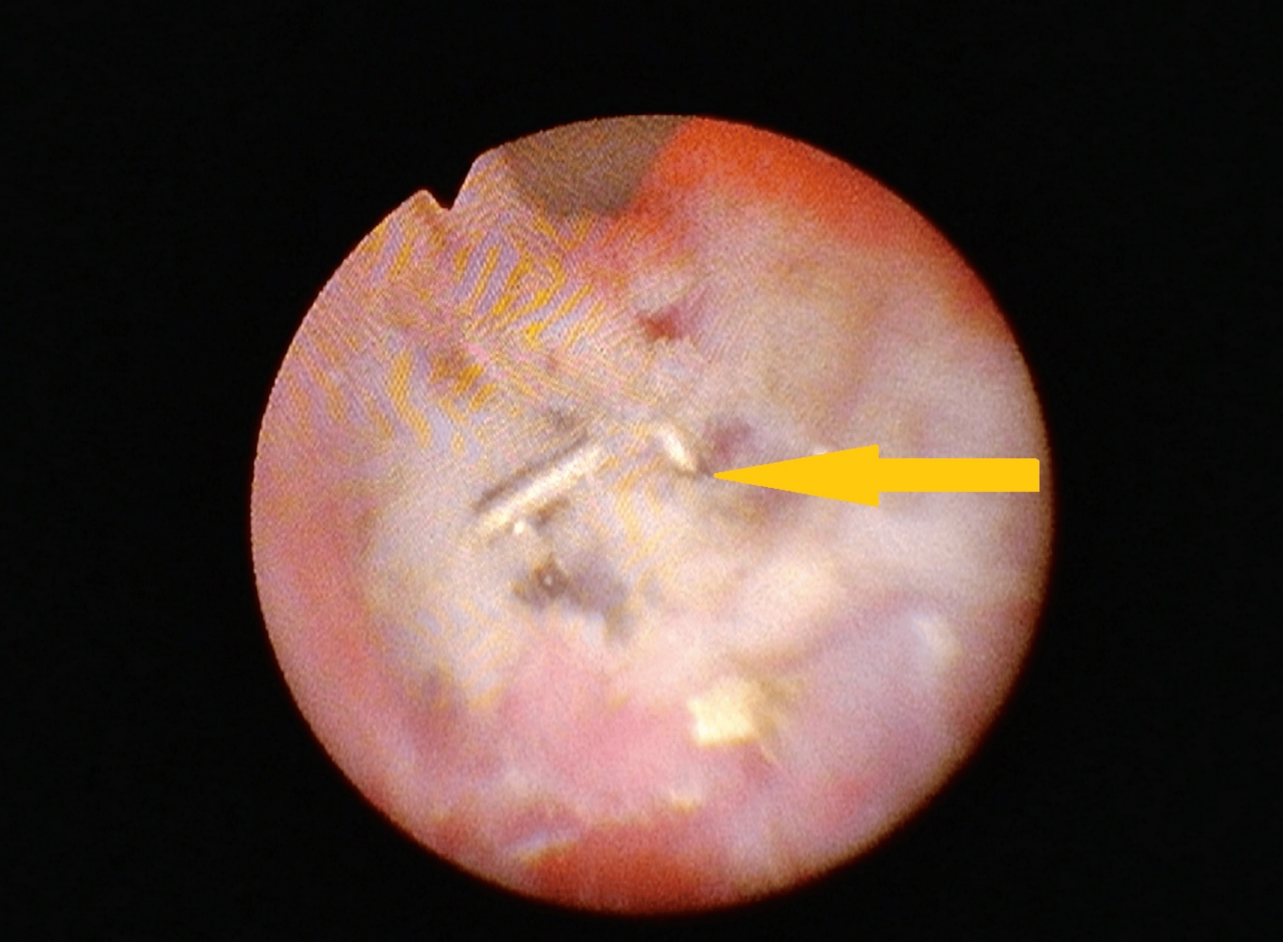
Endoscopic visualization of an Endo-GIA staple impacted in the mid-ureter GIA: Gastrointestinal anastomosis; Endoscopic image obtained during ureterorenoscopy. The yellow arrow highlights a metallic Endo-GIA staple embedded in the mid-portion of the left ureteral wall.

2) A left-sided mid-ureteral calculus (Figures 2, 3) associated with grade I hydronephrosis and proximal ureteral dilation.

The patient was referred to urologic evaluation. A 6 Fr double-J (DJ) (Vortek® Double Loop Ureteral Stent, Coloplast, Humlebæk, Denmark) ureteral stent was placed to relieve obstruction and decompress the upper urinary tract. Definitive hernia repair was deferred until after successful management of ureteral pathology.

**Figure 4:**
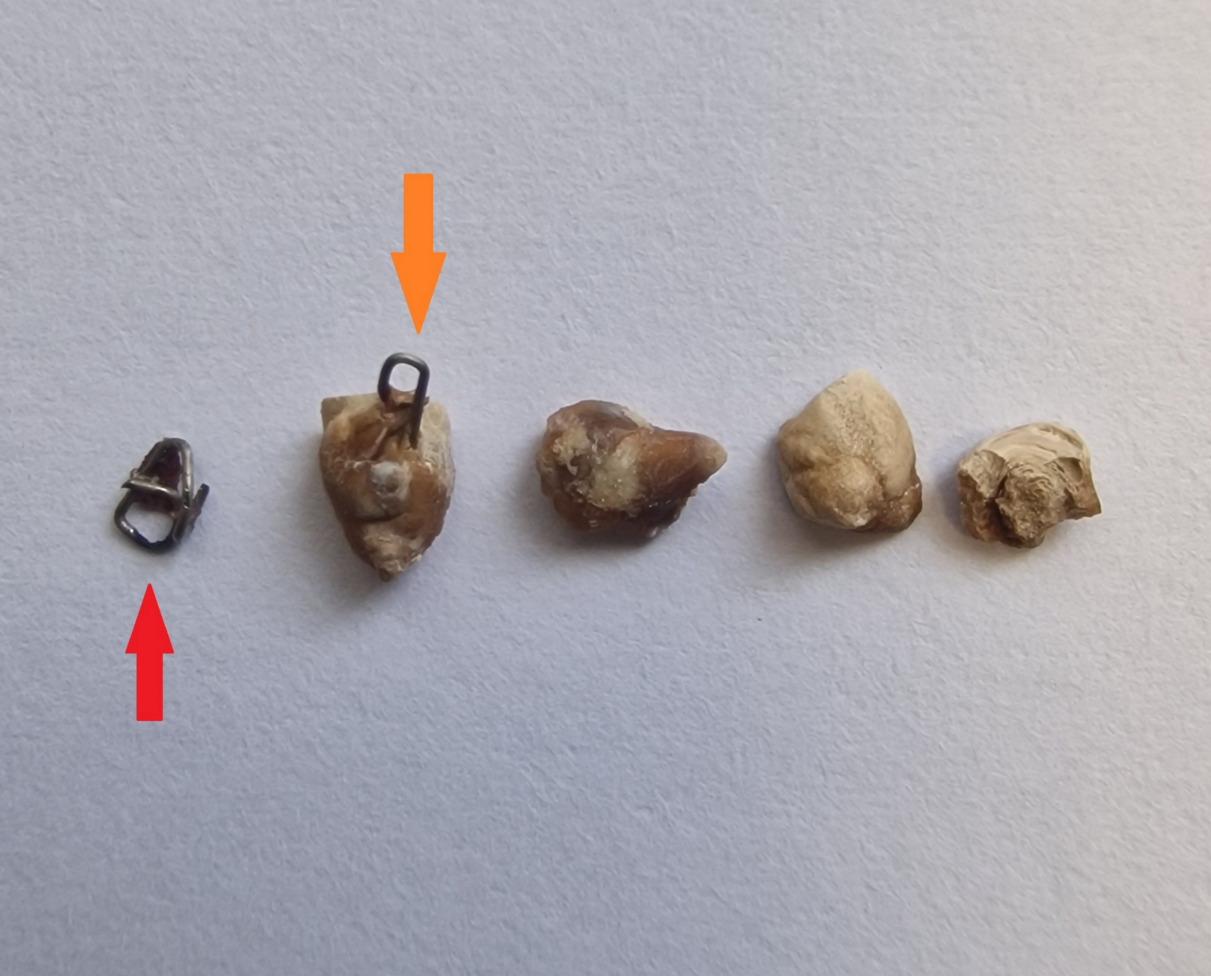
Macroscopic appearance of stone fragments and extracted Endo-GIA staples GIA: Gastrointestinal anastomosis; Macroscopic image showing the fragmented ureteral stone and two extracted Endo-GIA staples. The orange arrow marks a staple embedded within a stone fragment, while the red arrow indicates the staple removed from the ureteral wall.

The patient was subsequently admitted for planned endoscopic stone removal. Under general anesthesia, a rigid ureterorenoscopy (URS) was performed using thulium laser lithotripsy. Intraoperatively, the stone was noted to be firmly adherent to the ureteral wall, raising suspicion of a foreign body nidus. Upon partial stone fragmentation, a metallic staple was identified protruding through the ureteral mucosa (Figure 4).

**Figure 5:**
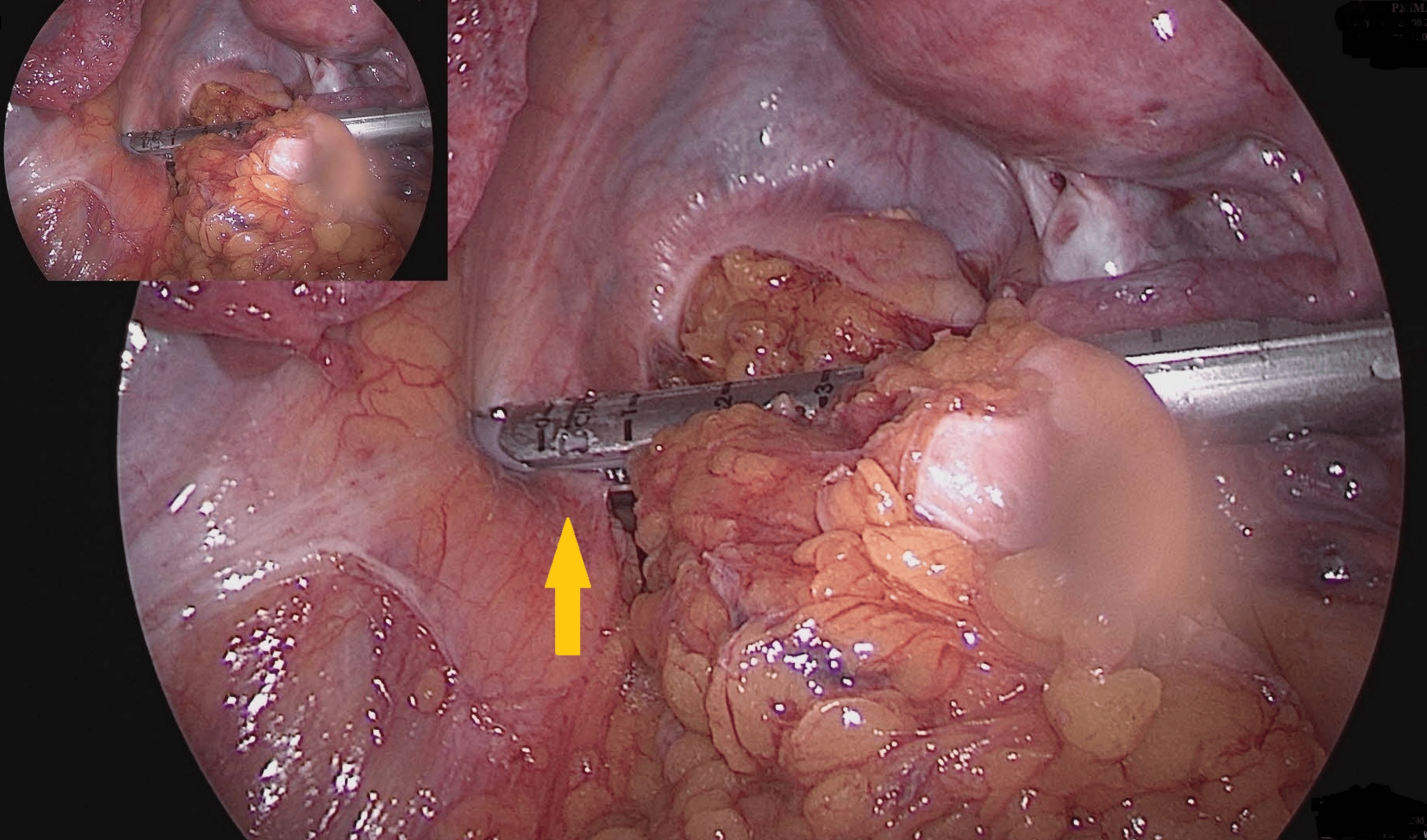
Intraoperative laparoscopic view during sigmoid resection The yellow arrow indicates the left ureter, which appears to have been unintentionally incorporated into the stapling line during transection.

It was carefully mobilized and removed using endoscopic graspers. Further inspection of the fragmented stone material revealed a second Endo-GIA staple fully embedded within the stone matrix, which was also extracted (Figure 5).

The affected segment of the ureter demonstrated signs of chronic mucosal change but no evidence of active perforation or extravasation. A new 8 Fr DJ stent (Vortek® Double Loop Ureteral Stent, Coloplast, Humlebæk, Denmark) was placed to ensure ureteral healing.

**Figure 6:**
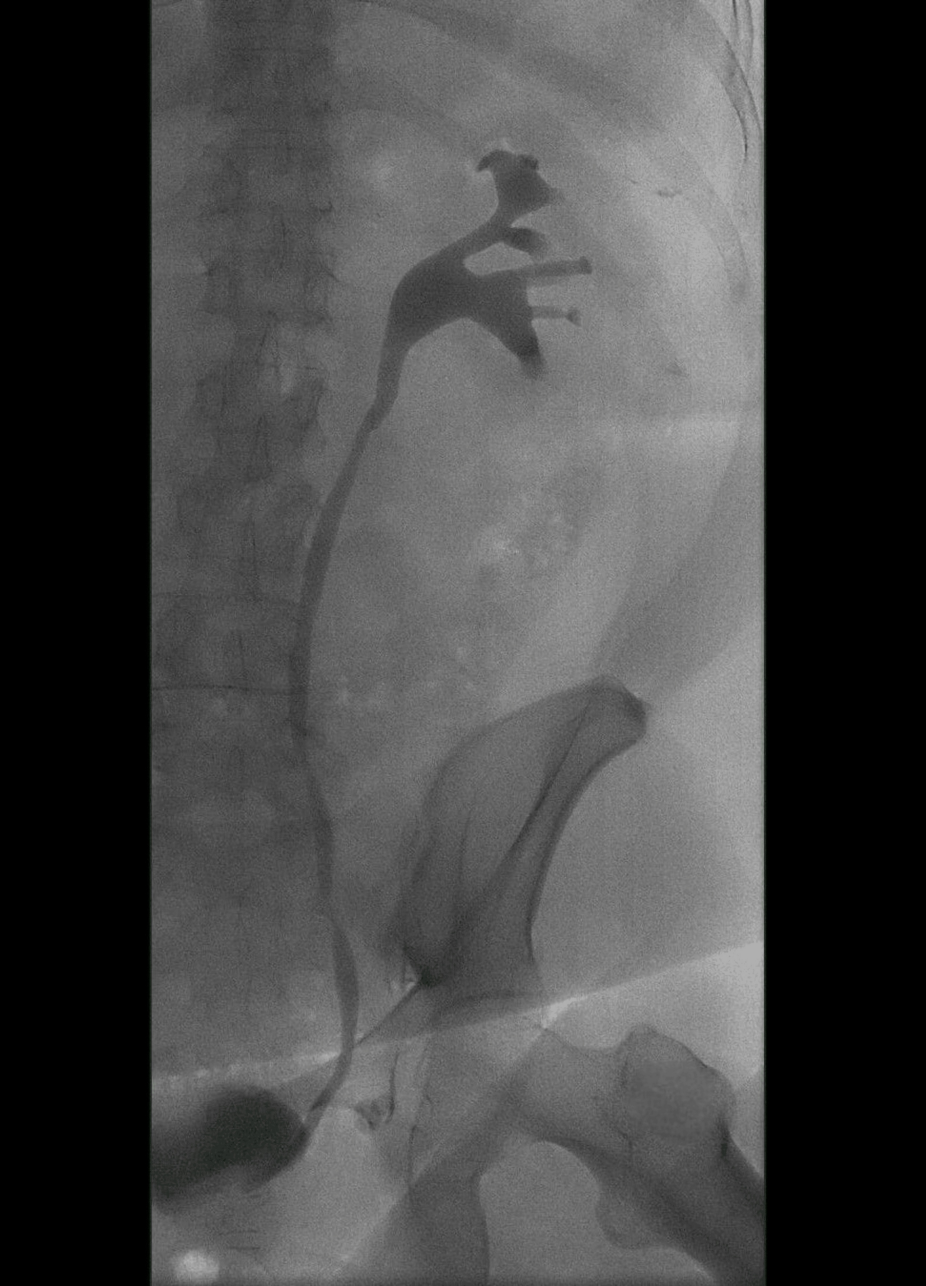
Retrograde ureteropyelography after stent removal Retrograde ureteropyelography six weeks after the stone and staple extraction, demonstrating a fully patent left ureter without stricture, obstruction, or residual filling defects.

These intraoperative findings were consistent with a delayed iatrogenic ureteral injury, likely caused by an unrecognized staple incorporation during the 2019 colorectal surgery. A subsequent search of archived intraoperative imaging identified a photo clearly showing partial ureteral inclusion within the stapler line (Figure 6).

The postoperative course was uneventful. The patient was discharged in good condition. At the six-week follow-up, the DJ stent was removed in the outpatient urology clinic. Follow-up imaging, including an intravenous urogram (Figure 6) and subsequent renal ultrasound, demonstrated complete resolution of hydronephrosis, and the patient remained clinically asymptomatic, confirming complete recovery. 

This case illustrates an uncommon and delayed complication of stapled colorectal surgery, ureteral obstruction due to retained surgical staples, successfully managed through endoscopic intervention with full functional recovery.

## Discussion

Mechanism of injury

Ureteral injuries during pelvic and colorectal surgery are uncommon but may result in serious complications such as urinary tract infection, long-term ureteral obstruction, and loss of renal function [1,11]. These injuries most often occur through direct transection, ligation, or thermal damage and are usually recognized intraoperatively or in the early postoperative period [12].

In this case, the laparoscopic sigmoid resection performed in 2019 was uneventful, and no ureteral injury was suspected at that time. Six years later, the patient presented with right-sided abdominal discomfort due to an incisional hernia, where imaging incidentally revealed a left-sided mid-ureteral stone with mild hydronephrosis but no urological symptoms. The stone was removed endoscopically by ureterorenoscopy and laser lithotripsy, during which two metallic staples were identified, one embedded in the ureteral wall and another enclosed within the stone matrix.

These findings suggested a chronic process resulting from partial incorporation of the ureter into the stapling line during the original surgery, which over time led to local irritation, inflammation, and subsequent stone formation. A retrospective review of archived intraoperative photographs confirmed inclusion of the left ureter within the stapler line, thereby confirming the presumed mechanism of injury. This case underscores the importance of systematic intraoperative photo and video documentation, which can provide crucial retrospective evidence when investigating rare, delayed surgical complications.

Role of foreign bodies in urology

The formation of urinary calculi around foreign materials is a well-recognized phenomenon in urology. Non-absorbable sutures, Hem-o-Lok clips, and retained ureteral stents have all been implicated in stone formation within the upper urinary tract [7-9]. In such cases, chronic mucosal irritation and prolonged contact with urinary solutes promote mineral deposition and encrustation, eventually leading to obstruction.

In the present case, two metallic staples were identified, one embedded within the ureteral wall and another completely enclosed in a ureteral stone. These served as nidus points for crystal aggregation and progressive obstruction through chronic local inflammation and mineral deposition.

Imaging and endoscopic management

Cross-sectional imaging, including CT, accurately localized the obstruction in the mid-ureter but failed to reveal the underlying foreign body, underscoring the diagnostic limitations of imaging when metallic staples are obscured within a stone matrix or soft tissue. Definitive diagnosis and treatment were achieved through URS. The use of thulium laser lithotripsy allowed precise fragmentation of the calculus while preserving ureteral integrity, and both staples were carefully retrieved with endoscopic graspers. The absence of ureteral perforation and the resolution of hydronephrosis after the DJ stent removal confirmed complete endoscopic resolution, thereby obviating the need for reconstructive surgery. This case highlights the effectiveness of endourological techniques in managing delayed ureteral complications associated with intraluminal foreign bodies [9].

Prevention strategies and literature evidence

This case underscores the importance of meticulous intraoperative ureteral identification, particularly in high-risk colorectal procedures involving inflammation, distorted anatomy, or previous surgeries. Several techniques have been developed to minimize the risk of iatrogenic ureteral injury. The use of intraureteral indocyanine green (ICG) has been shown to enhance real-time visualization of the ureters and reduce injury risk during complex robotic colorectal procedures [13]. A systematic review confirmed the safety and effectiveness of ICG-based methods but noted practical limitations, including the need for cystoscopy and intraureteral dye instillation [14].

Prophylactic ureteral catheterization has also been investigated as a means of improving ureteral identification. When performed simultaneously with surgical preparation, this technique facilitates intraoperative localization without significantly increasing operative time or complication rates [15]. The 2023 World Society of Emergency Surgery (WSES) guidelines recommend considering prophylactic stenting or fluorescence-guided techniques in patients with recognized risk factors such as obesity, prior pelvic surgery, or severe inflammation [2].

Similarly, a systematic review analyzing 26 studies concluded that both stent-based and fluorescence-guided methods are safe and feasible options for selected high-risk patients, although further randomized studies are needed to establish standardized recommendations for their routine use [16]. In the context of this case, such visualization techniques might have helped prevent inadvertent ureteral incorporation during stapler application.

Although not yet universally adopted, these techniques provide valuable tools to enhance intraoperative safety and may help prevent rare but significant complications such as the one presented in this report.

Clinical implications

This case offers new insight into a previously undocumented mechanism of delayed ureteral obstruction caused by retained stapler material and highlights several key considerations for pelvic surgeons. During laparoscopic procedures, especially in inflamed or fibrotic operative fields, meticulous identification and protection of the ureters are critical to prevent inadvertent injury [6]. Delayed iatrogenic complications should also be considered in the differential diagnosis of patients who present with new or unexplained urological findings years after stapled colorectal surgery. Moreover, endourological management remains a safe and effective therapeutic approach, even in complex cases involving intraluminal foreign bodies, and can often obviate the need for more invasive reconstructive surgery [8,9].

Limitations

No histopathological confirmation was available to definitively demonstrate a chronic inflammatory or foreign body reaction. As a single case report, the findings are inherently limited in their generalizability. Furthermore, the ureteral injury was neither recognized intraoperatively nor documented during the initial sigmoid resection, as the procedure and postoperative course were uneventful. The causal relationship could only be established retrospectively through review of archived intraoperative photographs, which revealed incorporation of the ureter into the stapling line. Despite these limitations, the case provides an important insight into a previously unrecognized mechanism of delayed ureteral obstruction and highlights the value of systematic intraoperative documentation.

## Conclusions

This case illustrates a rare but severe iatrogenic complication of stapled colorectal surgery, delayed ureteral obstruction caused by retained Endo-GIA staples. Such injuries are preventable and highlight the need for meticulous identification and protection of the ureter during pelvic dissection, particularly in inflamed or anatomically distorted operative fields. The case also emphasizes that the surgeon's expertise and careful attention to anatomical structures are critical for avoiding these complications. Although the injury remained clinically silent for years, endoscopic management proved safe and effective once diagnosed. Long-term awareness of possible late urological manifestations is essential when evaluating patients after pelvic surgery.
